# Application and development of aptamer in cancer: from clinical diagnosis to cancer therapy

**DOI:** 10.7150/jca.49532

**Published:** 2020-10-04

**Authors:** Jing Han, Liang Gao, Jinsheng Wang, Jia Wang

**Affiliations:** 1 Department of Immunology, Changzhi Medical College, Changzhi, Shanxi, 046000 China.; 2 Department of Reproductive Medicine, Heping Hospital Affiliated to Changzhi Medical College, Changzhi, Shanxi, 046000 China.; 3 Department of Dermatology, Heji Hospital Affiliated to Changzhi Medical College, Changzhi, Shanxi, 046000 China.; 4 Department of Pathology, Changzhi Medical College, Changzhi, Shanxi, 046000 China.

**Keywords:** aptamers, cancer, cancer diagnosis, targeted therapy, drug delivery

## Abstract

Traditional anticancer therapies can cause serious side effects in clinical treatment due to their nonspecific of tumor cells. Aptamers, also termed as 'chemical antibodies', are short DNA or RNA oligonucleotides selected from the synthetic large random single-strand oligonucleotide library by systematic evolution of ligands by exponential enrichment (SELEX) to bind to lots of different targets, such as proteins or nucleic acid structures. Aptamers have good affinities and high specificity with target molecules, thus may be able to act as drugs themselves to directly inhibit the proliferation of tumor cells, or own great potentialities in the targeted drug delivery systems which can be used in tumor diagnosis and target specific tumor cells, thereby minimizing the toxicity to normal cells. Here we review the unique properties of aptamer represents a great opportunity when applied to the rapidly developing fields of biotechnology and discuss the recent developments in the use of aptamers as powerful tools for analytic, diagnostic and therapeutic applications for cancer.

## Introduction

In recent years, many progresses have been made in treating diseases including cancer due to rapid development in many aspects. Globally, the high mortality rate of malignant tumors still critically endanger people's lives and health due to challenges arising in the clinical management and diagnosis of cancer 1, 2. Traditional tumor treatment methods include chemotherapy, radiotherapy and surgery, but these therapies have their own limitations in practical clinical applications. Surgical treatment is very effective for early solid tumors, but it is less effective for advanced tumors and unfortunately is not as good as non-solid tumors such as leukemia. Chemotherapy is currently an important method for treating tumors. Generally, it is distributed throughout the body after intravenous injection to achieve therapeutic effects. However, this treatment method lacks selectivity and will kill normal tissues while killing tumor tissues, causing non-specific damage. In addition, long-term use of chemotherapeutic drugs can cause patients to develop drug resistance and reduce the effectiveness of treatment. In radiotherapy, ionizing radiation is used to treat malignant tumors. Systemic and local radiotherapy often produces serious adverse reactions, such as radiation osteonecrosis, radiation pneumonia and systemic reactions 3, 4. Therefore, it is urgent to significantly reduce cancer mortality by exploring effective methods for cancer diagnosis, early detection and treatment strategies 5. The unique properties of aptamers that are compatible with multiple platform designs in the growing field of biotechnology have contributed to the rapid analysis, diagnosis and treatment of cancer 6.

Aptamers are single stranded DNA, RNA, or altered nucleic acids sequences with a strong affinity for specific target binding generated by the systematic evolution of ligands by exponential enrichment (SELEX) technology 7, 8. Aptamers have a unique three-dimensional spatial structure, which can bind to the target with high specific and affinity. Some aptamers have the function of regulatory proteins and can regulate the function of target proteins by binding to target proteins. Targets of aptamers are varied and include proteins, ions, cells, etc. 9, 10. Compared with antibodies, aptamers have the following advantages: stronger affinity with the target, higher specificity, easy preparation and modification, and good stability and easy to store 11. So far, a variety of aptamers targeting different targets have been screened. Due to the unique characteristics of aptamers, it has been widely used in detection, diagnosis imaging analysis and drug targeted therapy for tumor 12.

As synthetic molecules, aptamers modifications occur regularly and toward a specific purpose. Up to now, lots of modifications to aptamers have been applied to make them more versatile, such as modified with biotin, florescent dyes or radionuclides. In generally, the physical and biochemical properties of aptamer influence its biodistribution or pharmacokinetics 13. Therefore, to meet clinical purposes and improve the characteristics of aptamer, it is prerequisite to enhance aptamer's biocompatibility, stability and biovailability by grafting modification to improve its biochemical properties 14, 15. In addition, aptamers can efficiently deliver proteins, drugs or nucleic acids into specific structures in cells by conjugating to small interfering RNAs (siRNAs), drug molecules or nanoparticles, thereby reducing toxic and side effects 16-18.

Nano-sized particles or materials are made of biocompatible and biodegradable materials. It can efficiently deliver proteins, peptides or nucleic acids into specific cells 19. As a drug carrier, through the enhanced permeability and retention effect (EPR, that is, compared with normal tissues, molecules or particles of a specific size are more likely to accumulate in cancer tissues) of solid tumors, the nanomaterial can be non-specifically accumulated in the cancer tissue site 20-22. However, the passive targeting efficiency of the EPR effect is low. By combining aptamers that can specifically recognize antigens or biomarkers of tumor cell with nanomaterials, active targeted aggregation of nanomaterials in tumor tissue can be achieved and toxicity to normal tissues can be reduced 23. Recently, applications of aptamer-nanomaterial conjugates for cancer diagnosis and therapy have attracted increasing attention. Aptamers-nanomaterial complex with high affinity to tumor biomarkers are widely applied for the detection of circulating tumor cells (CTCs), providing early diagnosis and targeted therapy, opening a new choice for personalized medicine of cancer 24-26. Here, we summarize the development and applications of aptamers in cancer diagnostics and therapeutics and discuss an outlook of current problems and future perspectives in this field.

## Structure and properties of aptamers

The term “aptamer”, derived from the Latin 'aptus' meaning 'fit', was first reported in 1990 by Ellington et al. 27. Aptamers are short-stranded oligonucleotides, that is, single-stranded DNA or RNA sequences, which can bind specific targets and have great potential for targeted diagnosis and treatment of tumors 28. Aptamers usually fold into a unique three-dimensional structure and can specifically recognize targets such as small organic molecules, proteins, and cells 29-31. The three-dimensional structure of an aptamer is characterized by stem, inner ring, bulge, hairpin, pseudoknot, triplicate or G-quadruplex structure. The combination of aptamer and its target is due to the complementarity of the combined geometry, the stacking interaction of the aromatic ring with the aptamer nucleobase, the electrostatic interaction between the charged groups, the van der waals interaction, and the hydrogen bond 32. Aptamers have received increasing attention since their discovery and are used as diagnostic and therapeutic targeting ligands 33-35. Compared with antibodies, it has a small molecular weight, stable structure, plasticity of chemical groups, fast blood clearance, and non-immunogenicity 36 (**Table 1**). Studies have shown that the dissociation constant of some RNA aptamer-target complexes can reach picomolar levels, showing that the aptamers have stronger affinity for binding targets than antibodies. Aptamers have high specificity, and can strictly recognize the difference between a target molecule and non-target molecules or even a group and different amino acids 37. Aptamers as therapeutic drugs and targeted ligands for drug delivery, binding with cancer cells and related protein targets can improve the efficacy of tumor treatment and reduce toxic and side effects.

## Screening of aptamer

**Figure 1:**
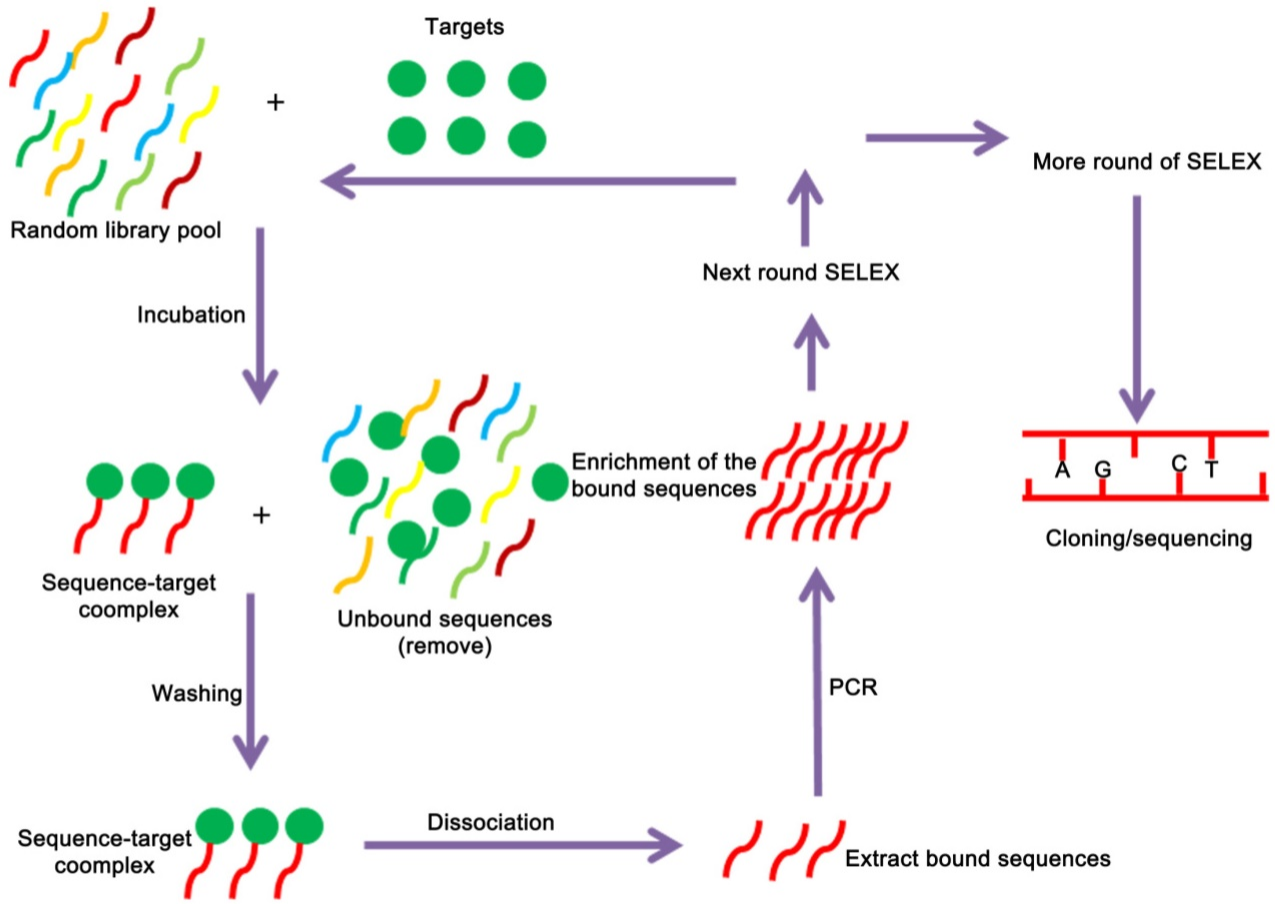
** A schematic representation of the SELEX strategy of aptamer selection.** The targets are used to screen and enrich the random DNA oligonucleotide library pools, and cloning and sequencing are performed to obtain the fittest aptamers.

Aptamers are selected by SELEX technology, which begins with a chemically-synthesized oligonucleotide library that contains 10^13^-10^16^ different sequences 38. The SELEX technology mainly consists of three steps: First, construct a random screening library of DNA or RNA *in vitro* by molecular biology technology, and then incubate the random library with the target to isolate the binding sequence. After that, the PCR amplification product can be used for the next round of screening, which can be enriched after several rounds of screening. A nucleic acid library with high affinity to the target is collected, and a specific aptamer sequence can be obtained after cloning and sequencing 39, 40 (**Figure 1**). Although the conventional SELEX can effectively screen aptamers *in vitro*, a typical SELEX process can take weeks or even months. In recent years, with the emergence of various new, efficient and high-throughput screening methods, including automation technology, capillary electrophoresis technology, microfluidic chip technology, nanotechnology, high-throughput sequencing technology, etc., there has been significant development both in in screening objects and screening efficiency 41-44. For example, capillary electrophoresis SELEX technology which has the characteristics of reduced sample injection, high separation efficiency, economy, and high degree of automation. It is widely used for material separation and analysis. In 2004, Bowser's research group firstly introduced capillary electrophoresis technology to the screening of aptamers. Capillary electrophoresis technology can efficiently separate the bound and unbound aptamer molecules with only a few round screening, which has greatly improved the screening efficiency and greatly shortened the screening cycle 45. Berezovski et al. further improved capillary electrophoresis SELEX by constructing a “non-equilibrium capillary electrophoresis of equilibrium mixtures (NECEEM)” for screening aptamers. The biggest advantage of this method is the low background, which is 100 ~ 1000 times lower than that of the traditional method 46, 47. Besides the capillary electrophoresis SELEX that tailored or primer free SELEX 43, 48, toggle SELEX 49, expression cassette SELEX 42, photo SELEX 50 and automated SELEX 51 have also greatly improved the selection and screening of aptamers.

In addition, cancer cells are characterized by cell surface molecules that are over-expressed or altered due to multiple oncogenic mutations, so scientists have further developed cell-SELEX aptamer screening technology 52, 53 (**Table 2**). The cell-SELEX screening process is similar to traditional SELEX, including incubation, isolation, and amplification. Unlike traditional SELEX targeting individual target molecules, the target of cell-SELEX is intact living cells, retaining the natural conformation of cell surface proteins, and the screened aptamers are more suitable for biological applications. Since there is no need to first detect the molecular markers on the cell surface, it brings great convenience to the aptamer selection process 54, 55.

## Modification of aptamer

**Figure 2:**
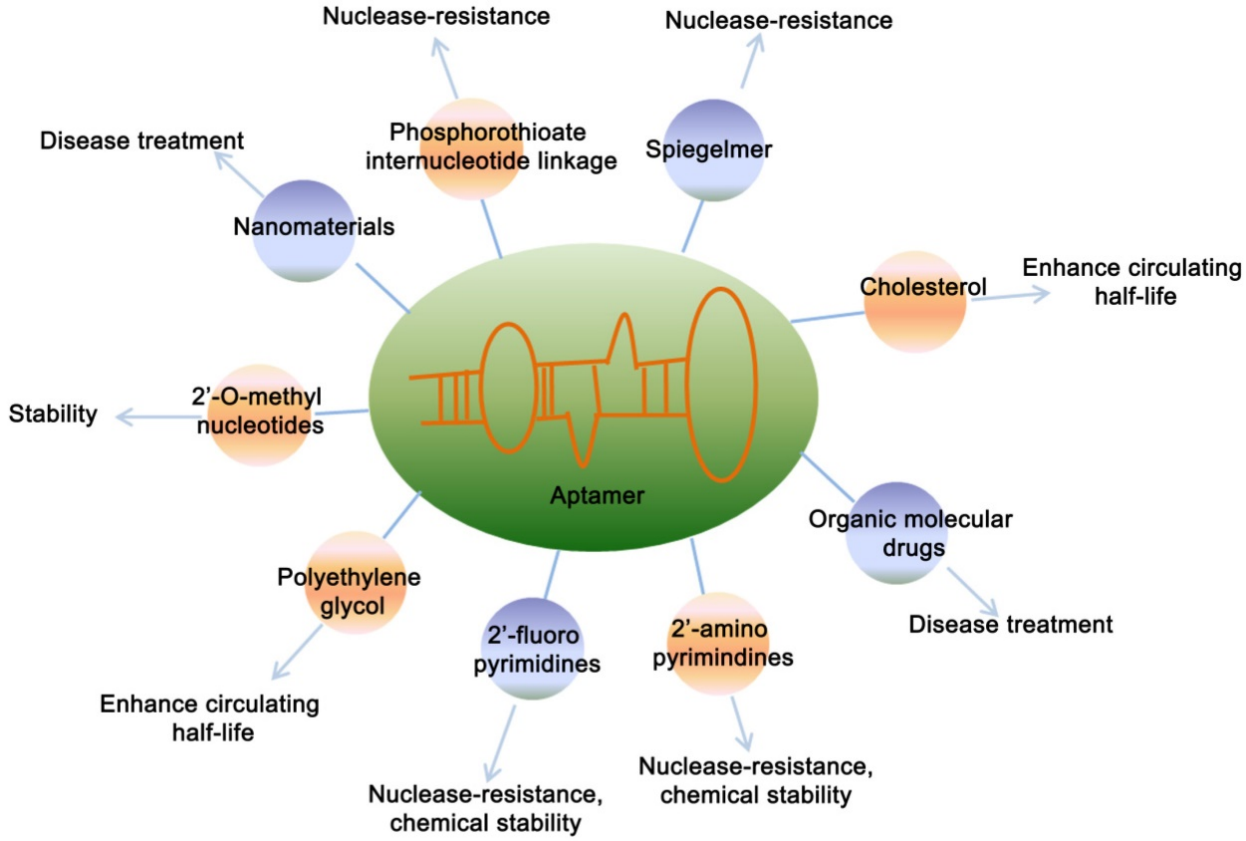
** Chemical modifications of aptamers.** Figure represents the various modifications of aptamer to increase its stability and potential applications.

Considering the wild-type aptamer molecules have very short half-life due to the clearance from the body by kidney and nuclease-mediated degradation, which limited their application under physiological conditions. Therefore, a number of modified aptamers through biochemistry approaches have been used to overcome aptamer's instability and optimize the pharmacokinetic and pharmacodynamic properties of aptamer 56 (**Figure 2**). Specific modifications facilitate the delivery aptamers into target cells with precised specificity. Some modifications of aptamers by functional optimization, multimerization or truncation have been shown to enhance the stability and binding efficacy. Various modification designs, conjugation strategies and linkage approaches are prevalent in aptamer technology 56. Chemical modification of aptamers is common to enhance its stability and functionality. A strategy is to use a pool of oligonucleotides with the chemical substitutions already exist in SELEX process. When amplified, aptamers can be generated which are partially or completely substituted with one or more modifications, including 5´-bromide 57, 2'-amino 58, 59, 2'-fluoro 18, 60, 61, 2'-O-methyl nucleotides 62, 63, cholesterol 64, 65 and polyethylene glycol (PEG) 66 etc.

In recent years, chimeric aptamers have become an insight because of their greater stability and nuclease resistance. Chimerization of aptamers refers to its combination with another aptamer, siRNA, protein, enzyme, biomacromolecules, drug, imaging agents or dyes 67. The aptamers chimerization, with two or more different chemical or biological components, provides great plausibility for engineering new multifunctional aptamers-based structures 68.

With the progress of research in this field, it is very clear that chimeric aptamers are not only highly stable and efficient but are also able to deliver drugs. For instance, The anticancer aptamers chimera systems are able to specifically bind to tumor cells and deliver their drugs to the target cells 69. Aptamers chimera systems also can be designed with the capability of concurrent binding to cancer cells and lymphocytes, which can induce an enhanced antitumor and cellular immunity by simultaneous targeting of cancer cells and the immune cells 70.

## Applications of aptamers in cancer

Most of the traditional anti-tumor drugs are not selective and have caused serious toxic and side effects in clinical treatment 71. Aptamers are a class of small nucleic acid ligands that have high affinity and specificity for their targets 7. Aptamers screened by using biomarkers closely related to the development of cancer as targets can be used as drugs themselves, and can also act as a targeted drug delivery system by conjugating with drugs, siRNA, nanoparticles, etc. to form a targeted drug delivery system which can target specific tumor cells, thus minimizing the toxicity to normal cells, reducing the dose needed for treatment and enhancing therapeutic efficacy.

Currently, a variety of aptamers targeting tumor cells have been screened by SELEX; for example, A10, anti-prostate-specific membrane antigen (PSMA) aptamer 72, AS1411, anti-nucleolin aptamer 73, 74, EpCAM, anti-epithelial cell adhesion molecule aptamer 75, 76, Sgc8, anti-protein tyrosine kinase 7 (PTK7) aptamer 77, 78, MUC1, anti-mucin1 aptamer 79, etc. Moreover, for these aptamers, a variety of drug delivery systems have been developed for targeted treatment of specific tumor cells.

## Roles of aptamers for biosensors

In recent years, aptasensors, a biosensors that use aptamers as bio-receptors, have been attracted noticeable attention for cancer biomarker detection 80. Most biosensors are designed based on the principle of antibody-antigen binding 81. Two different types of antibodies are used to detect target molecules. However, due to the different affinity of these antibodies, the use of biosensors for disease diagnosis has caused problems. Compared with antibodies, aptamers have many excellent properties, the conformation of the aptamer changes after binding to the target molecule, which provides the possibility to design unique and variable sensors. Therefore, aptamers, as a promising biosensor, have been used in the design of tumor-related biomarker sensors, including aptamer-based optical sensors, electrochemical sensors, etc. 82-85.

Aptamer-based optical sensors had made great improvements in a variety of fields such as life science, medicine and environmental monitoring 86. Among aptamer-based optical techniques, mainly including colorimetric based biosensors 87, surface enhanced raman scattering (SERS) based optical biosensors 88, fluorescent based optical biosensors 89, luminescent based optical biosensors 90, chemiluminescence based optical biosensors 91 and electrochemiluminescence based optical biosensors 92. For example, Hu et al. used fluorescein isothiocyanate (FITC) labeled aptamer as energy donor to construct a highly sensitive fluorescence resonance energy transfer (FRET) aptasensor for sensing immunoglobulin E (IgE) 84.

Aptamer-based electrochemical sensors, as an important sub-group of sensors, are highly attractive and applicable for diagnosis of cancer and use in point-of-care tools, in addition to multi-analyte detection 93. Among aptamer-based electrochemical techniques, mainly including amperometric, impedance, surface charge using field-effect transistors (FETs) and voltammetric sensors based on electrochemical transduction 94. For example, Liao et al. used nanomaterials as a sensing probe for construction of an electrical impedance spectroscopy (EIS) electrochemical aptasensor for the detection of platelet-derived growth factor (PGDF), which is an important protein biomarker of cancer. They synthesized Co3(PO4)2-based nano-complex through a simple self-assembly method aided by aptamer and BSA. These composite could detect efficiently PDGF concentrations as low as 3.7 pg/mL 95.

## Roles of aptamers in cancer diagnostics

Cancer is the second leading cause of death worldwide 96, 97. Accurate tumor diagnosis technology has positive clinical significance, which can help doctors to propose treatment strategies early, evaluate treatment effects, monitor tumor recurrence and metastasis, and assess prognosis. At present, antibodies are widely used in clinical diagnosis of tumors, such as flow cytometry, tumor marker detection, immunohistochemistry, *in vivo* imaging, etc. 98. However, due to the shortcomings of antibodies such as high immunogenicity, poor stability, difficult chemical modification, limited production methods and expensive production costs, their clinical applications have been limited to a certain extent. Compared with antibodies, aptamers also have the ability of high affinity, high specificity to bind to targets, and have obvious advantages in chemical modification, stability, and production cost. It has been widely used in various fields of tumor diagnosis, such as circulating tumor cells (CTCs) detection, immunohistochemical analysis, and *in vivo* imaging.

## Aptamers for cancer detection

Sensitive detection of cancer cells plays an important role in in cancer diagnosis and prognosis 99. At various stages of tumorigenesis and development, some specific tumor markers, such as CTCs, produced or secreted by tumor cells into the blood system are good targets for liquid biopsy 100. Accurate and efficient detection of CTCs in the early stages of cancer when the concentration of CTCs is low will significantly help monitor the patient's condition and progression of cancer 101. However, CTCs detection is highly challenging field due to very few cancer cells in blood compared to very majority blood cells 102, 103. In recent years, a series of analysis methods based on aptamers have been applied to CTCs detection by combining or conjugating different signal reporting technologies such as fluorescence, colorimetric, magnetic, and electrochemical technologies 104-107. For example, Karnik et al. developed a platform to isolate and capture CTCs using a DNA network comprising repeating adhesive aptamer domains 108. Kashefi-Kheyrabadi et al. established an electrochemical technique for liver cancer HepG2 cell-specific aptamer conjugated to a gold plane electrode for CTCs detection of liver cancer 105. Fan et al. developed a platform linking multivalent DNA aptamer nanomaterials with microfluidic devices for isolating of cancer cells from blood 109. Aptamer-functionalized nanostructures have also been developed for cell catch and isolate. Wang et al. established a dual-aptamer-targeted high-sensitivity CTCs detection platform, conjugating VEGF aptamers with magnetic beads for capturing and concentrating CTCs, and then using MUC1 aptamer-conjugated Pt-Au nanoparticles. The nanoparticles were incubated with concentrated CTCs. In the presence of TMB (3', 3', 5', 5'-tetramethylbenzidine) and hydrogen peroxide (H_2_O_2_), Pt-Au nanoparticles quickly catalyzed the colorimetric reaction and transmit a sensitive signal. The system has a detection limit of 10 cells/mL and a linear range of 10 to 10^5^ cells/mL 107.

## Aptamers for cancer imaging

Molecular imaging is of great significance in disease detection, surveillance and prognosis 110. By combining fluorescent molecules 111, 112, radionuclides 113 or other imaging molecules 114, aptamers are becoming important tool for cancer diagnosis. *In vivo* imaging technologies for tumor diagnosis include luminescence imaging, computed tomography (CT), magnetic resonance imaging (MRI), radionuclide-based positron emission tomography (PET), and single photon emission computed tomography (SPECT). For example, IRD800CW labeled CD30 aptamer is used for *in vivo* imaging of lymphoma 115, Cy5 labeled pancreatic cancer-specific aptamer is used for *in vivo* imaging of pancreatic cancer 116, AS1411 aptamer coupled with blood-brain barrier targeting peptide for *in vivo* imaging of gliomas 117), etc. All of them show fast and specific tumor targeting ability, high signal-to-noise ratio and good pharmacokinetic properties. In addition to imaging with fluorescent molecules, aptamers can be conjugated to magnetic beads to enhance MRI 114. Such as vascular endothelial growth factor receptor 2(VEGFR2) aptamer conjugated with magnetic nanomaterials for glioma MRI imaging 118.

Epidermal adhesion molecule (EpCAM) aptamer conjugated with magnetic nanomaterials for MRI imaging of gastric cancer 119, etc. All significantly improve the tumor-targeted imaging capability, sensitivity and biocompatibility, and reduce cytotoxicity, further improving the clinical application potential of MRI.

## Aptamers for immunohistochemical (IHC)

In recent years, aptamers have been widely used in IHC analysis of tumor tissues, and have shown some application characteristics superior to antibodies. Zu's group performed IHC analysis of formalin-fixed-paraffin-embedded lymphoma samples and found that the CD30 aptamer showed almost the same staining pattern as the CD30 antibody, but its reaction conditions were simpler than antibody. Such as antigen retrieval temperature is 37 °C (CD30 antibody is 95 °C), incubation time is 20 minutes (CD30 antibody is 90 minutes). In addition, unlike antibodies, aptamers do not cause non-specific staining of necrotic areas in tissue samples when applied to IHC 120. In addition, Li X et al. screened a group of aptamers that can specifically identify metastatic lymph node tissues of colon cancer, that is, using aptamers as probes, immunohistochemical analysis of different type of tumor tissue samples and found that aptamers can specifically identify colon cancer tissues with lymph node metastasis and lymph node tissues with colon cancer metastasis, but no signal was reported in non-metastatic colon cancer tissue samples or other control tissues, suggesting that the that the target recognized by the aptamer is related to the process of colon cancer metastasis and can be used as an early diagnostic tool for colon cancer 121.

## Roles of aptamers for cancer therapy

**Figure 3:**
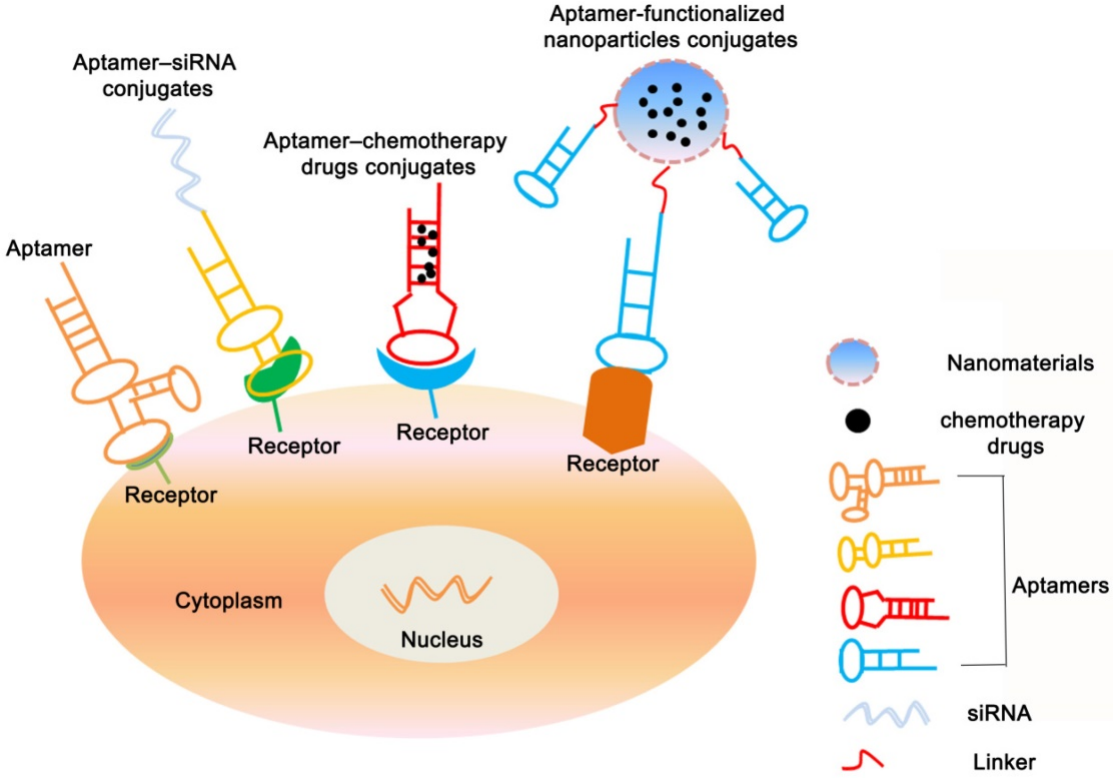
** Applications of aptamers-based for cancer therapy.** The figure shows that in addition to being used as a therapeutic drug, aptamers can also be congujated with siRNA, chemotherapy agents and nanopaticles to kill cancer cells.

At present, many chemical drugs can effectively kill cancer cells, but also destroy normal tissue cells and cause serious adverse reactions. Therefore, targeted drug delivery is the key to current tumor treatment. Aptamers have become a new direction for tumor-targeted drug therapy and an ideal tool for therapeutic applications due to their unique physical and chemical properties 35, 122. Such as therapeutic aptamers, aptamer-drug conjugates (AptDC), aptamer-functionalized nanoparticles, and aptamer-mediated immunotherapy (**Figure 3**).

## Therapeutic aptamers for cancer therapy

With development of aptamers selected from Cell-SELEX, an increasing number of aptamers are capable for use as therapeutic drugs for disease (**Table 3**). For example, the first aptamer targeted to human VEGF for the treatment of age-related macular degeneration was approved by food and drug administration (FDA) in 2004 123. Recently, aptamers were also developed as therapeutic agents for cancer treatment. The aptamer AS1411 (developed by Antisoma) 124, 125 and olaptesed pegol (also known as NOX-A12, developed by NOXXON Pharm) 126, 127 are currently in the phase II clinical research stage. AS1411, an unmodified DNA aptamer with G-quadruplex structure known as a non-SELEX aptamer that binds to nucleolin, was discovered serendipitously by Bates et al. 124. AS1411 has shown growth-inhibitory functions against a broad range of cancer cells *in vitro* 128. AS1411 could also link with nuclear factor-κB (NF-κB) to inhibit its activity and destabilize BCL-2 mRNA that all can suppress cell proliferation 129. Compared with monovalent aptamers, multivalent aptamers have stronger antitumor activity because of they can further increase the affinity with the target, multimerize the receptor, and activate downstream signals. Recently, Mahlknecht et al. screened a HER2 aptamer and constructed its trivalent structure. The results showed that the trivalent HER2 aptamer had 2 times higher antitumor activity than the HER2 antibody 130. The results of Parekh et al. also showed that the trivalent CD30 aptamer has stronger anti-tumor proliferation ability 131.

## Aptamer-based conjugates for cancer therapy

Besides the direct therapeutic effect of aptamers for cancer, most of the aptamer-based investigations for cancer treatment focus on the specific targeting ability to different cancer cells. As a result, A series of aptamer-drug conjugates (AptDC) models have also been successfully established in recent years and are undergoing extensive preclinical evaluation 132.

## Aptamer-siRNA conjugates

Gene drugs such as siRNA and microRNA also have important clinical application value. However, the lack of targeting and inability to effectively enter tumor cells has greatly reduced the feasibility of its clinical application. Conjugating with aptamers can increase many application advantages of gene drugs, such as making it obtain tumor targeting ability, and improving the efficiency of entering cells by receptor-mediated internalization pathways. In recent years, many aptamer-conjugated gene drug models have been established, and extensive preclinical evaluations have confirmed their feasibility for tumor treatment, such as PSMA aptamer conjugated with Plk1 or Bcl2 siRNA for prostate cancer treatment 18, HER2 aptamer conjugated with Bcl2 siRNA and cisplatin for breast cancer treatment 133, MUC1 aptamer conjugated microRNA-29b for ovarian cancer treatment 134 et al.

## Aptamer-chemotherapy drugs conjugates

Chemotherapy is primary approach for cancer treatment. However, the shortcomings of chemotherapeutic drugs are inevitable owing to serious toxic side effects, such as their poor water solubility, nonspecific distribution and systemic toxicity greatly reduce delivery efficiency, which limits their use in clinic. Up to now, targeted chemotherapy is vital to avoid its side effects and enhance its therapeutic efficiency 135. Aptamers conjugated with chemotherapeutic drugs as a kind of targeted therapeutic mean would increase drugs delivery effect and reduce cytotoxicity of drugs in normal tissue. For instance, Bagalkot et al. specifically delivered doxorubicine (Dox) into LNCaP cells through aptamers' binding capability to the prostate-specific membrane antigen (PSMA) on LNCaP cells 16. CD117 aptamer covalently coupled with methotrexate for the treatment of acute myeloid lymphoma 136. Protein tyrosine kinase 7 (PTK7) aptamer covalently coupled with Dox for treatment of lymphocytic leukemia via an acid-sensitive linker 137, etc. These conjugates have shown good specificity of tumor targeting ability, fast drug release efficiency, significantly reduced off-target effects, and significantly improved therapeutic effects in both *in vivo* and *in vitro* studies. In addition to covalent coupling, for some aptamers containing consecutive paired GC/CG sequences in a three-dimensional structure, anthracycline antitumor drugs (such as Dox) can be incorporated by non-covalent binding in the GC/CG structure of aptamers, forming a simple and effective AptDC model, such as epidermal adhesion molecule (EpCAM) aptamer coupled with doxorubicin for the treatment of retinoblastoma 138 and ligand-coupled doxorubicin for the treatment of breast cancer 139.

## Aptamer-functionalized nanoparticle conjugates

Due to the small molecular weight of the aptamers, the half-life in the blood circulation is short, which seriously affects the efficacy. Nanomaterials with a certain range of diameters can target to tumor cells via the enhanced permeability and retention (EPR) effect. However, this way of increasing drug enrichment through the EPR effect is still a “passive” targeting mode, which is susceptible to some factors such as structural differences in new blood vessels, blood pressure, and the pathological type and location of tumors, resulting in significant individual effects difference 140. Combining aptamers with nanomaterials can not only effectively extend the half-life in blood circulation, but also the large specific surface area of nanomaterials can also increase the load of drugs and aptamers, and the uniform morphology also makes them exhibit good biological distribution. Therefore, aptamer-nanoparticles are a targeted drug delivery system with promising applications 141-146. In recent years, different inorganic or organic nanomaterials have been developed including gold nanomaterials, magnetic nanomaterials, single-walled carbon nanotubes, silica nanoparticles (MSNs), quantum dots, liposomes, copolymers, and nucleic acid-based and protein-based nanomaterials. A series of aptamer-targeted nanodrug models have been successfully constructed 147 (**Table 4**).

Due to the special photothermal effect of gold-silver nanoparticles, Huang et al. conjugated it with the aptamer sgc8c that specifically recognizes CCCRF-CEM cells to form an Apt-NPs complex, which can be used for photothermal treatment. The results show that the Apt-NPs complex can not only target tumor cells, but the temperature of AuNPs will continue to increase under near-infrared light to kill tumor cells, but it will not cause damage to normal cells (survival rate is 87%), reduced required laser exposure, and greatly reduce the amount of laser irradiation required 148.

Zhang et al. established an aptamer-targeted nanodrug co-delivery system. In this system, a PSMA aptamer containing a GC/CG repeat is used to assemble the hydrophilic drug doxorubicin and a polylactic acid-glycolic acid copolymer (PLGA) is used to encapsulate the hydrophobic drug docetaxel. After the drug assembly is completed, the aptamer and PLGA nanoparticles are coupled by PEG molecules. *In vitro*, the drug co-delivery system can specifically target PSMA-expressing prostate cancer cells and show significantly higher tumor killing activity than single drugs 143.

Pala et al. constructed dextran-encapsulated magnetic nanomaterials, which were combined with DNA aptamer that specifically recognized HER2 for magnetic hyperthermia of tumor cells. The results show that this system can highly specifically target SK-BR3 cells over-expressed by HER2, and 50% of SK-BR3 cells can be killed by magnetic hyperthermia. The therapeutic effect is 90 times that of magnetic nanomaterials alone. Moreover, the survival rate of HER2-negative U-87MG cells is close to 100%, which greatly reduces the dose of magnetic nanomaterials required for traditional magnetic hyperthermia and further reduces the side effects of treatment 149.

## Aptamer-based immunotherapy

**Figure 4:**
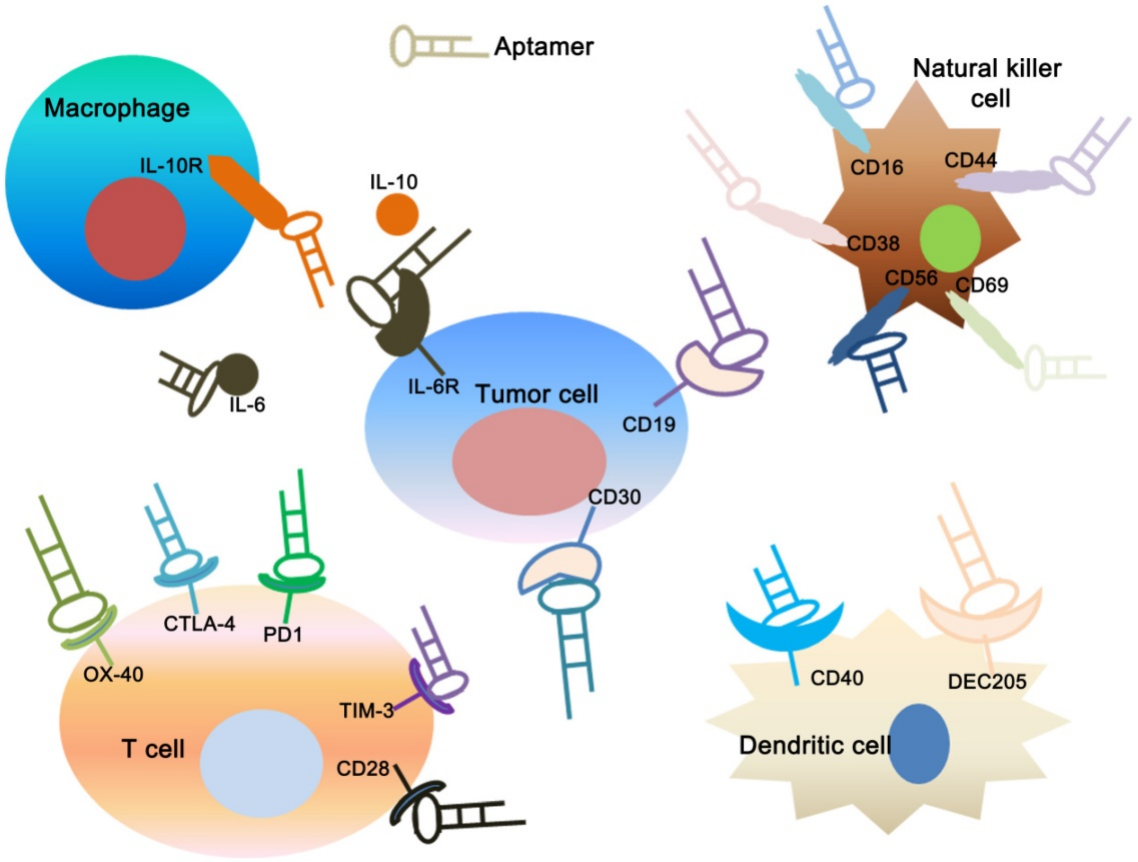
** Applications of aptamers in cancer immunotherapy.** The figure shows that specific aptamers that target molecular markers on the surface of tumor cells, dendritic cells, T cells, natural killer cells and macrophage cells can be used for tumor immunotherapy.

As a relatively novel method for cancer therapy, cancer immunotherapy has become increased attention due to its high specificity and low side effects, even rivaling other standard treatments 150. There are many cancer immunotherapy strategies, such as vaccine-based therapies, cell-based therapies and cytosine-guanine oligodeoxynucleotide (CpG)-based therapies 151, 152. In recent years, many aptamer constructs have been described that can regulate the immune response against cancer 153 (**Figure 4**). They provide a similar or even superior ability to that of the corresponding monoclonal antibody, and their superior targeted delivery ability confers on them less off-target side effects 154. Herrmann et al. used CTLA-4 aptamer conjugated gene drug STAT3 siRNA (CTLA4apt-STAT3 siRNA) to establish an innovative immune checkpoint gene therapy method. The results show that this method can significantly activate anti-tumor immunity and suppress tumors growth and metastasis 155. CTLA4apt-STAT3 siRNA can lead to internalization into tumor-associated CD8+ T cells and inhibit the expression of STAT3, which can activate the tumor antigen-specific T cells. Furthermore, CTLA4apt-STAT3 siRNA can dramatically reduce tumor-associated Tregs. In addition, Zhang et al. used nature killer (NK) cells as anti-tumor immune cells to eliminate residual tumor cells after photothermal therapy (PTT), and the NK cells were modified by TLS11a aptamer against hepatocellular carcinoma (HCC) cells on the cell surface to enhance immunotherapy efficiency 156. PD-1 is expressed in some cell types including T cells, specifically in CD8 tumor-infiltrating lymphocytes (TILs) which are responsible for killing tumor cells 157. Prodeus et al. screened a mouse-derived PD-1 aptamer that can specifically block the binding of PD-1 and PD-L1, thereby reversing the immunosuppressive state of the tumor and activating anti-tumor immunity. In the tumor-bearing models of PD-1 positive colon cancer, PD-1 aptamers can significantly inhibit tumor growth, and the treatment effect is similar to that of PD-1 antibodies 158.

## Conclusion and perspectives

Aptamer, a novel specific combining tool to various types of target, has attracted an increasing attention for cancer diagnosis and therapy. In this review, we review recent advances in this promising field of aptamer, including the screen of aptamer by SELEX process, modification of aptamers and applications of aptamenr for biosensing, bioimaging and therapy in cancer. Although aptamers have made great progress in tumor application research, they still need to continue to improve of drug loading rate, targeting efficiency, circulation time, and affinity, etc. The combination of aptamers and drugs and the modification of nanocarriers by aptamers still need to be improved. With the continuous advancement of SELEX technology and chemical modification methods, we believe that aptamers will definitely play a more and more important role in future oncology applications.

## Figures and Tables

**Table 1:** Comparison the characteristics of aptamers and antibodies

Characteristics	Aptamers	Antibodies
Targets	Widely	Mostly immunogenic macromolecular targets
Size	15~30 kD	50~100 kD
Affinity	High	High
Immunogenicity	Low	High
Tissue penetration	Fast	Slow
Production process	Chemical	*In vivo* production
Stability	High	Low
Toxicity	Low	High
Cost	Low	Expensive

**Table 2:** Aptamers targeting markers of cancer cells

Aptamers	Targets	Target cells	References
SYL3C	EpCAM	MCF7 (breast cancer)	159
A15	CD133	Hep3B (liver cancer)	160
Apt928	CD70	SKOV-3 (ovarian cancer)	161
A10	PSMA	LNCaP (prostate cancer)	162
GBI-10	Tenascin-C	U251 (glioblastoma)	163
Apt1	CD44	A549 (lung cancer)	164
KH1C12 aptamer	KH1C12	HL-60 (leukemia)	165
CD24 aptamer	CD24	HT-29 (colon cancer)	166
TD05	IGHM	Ramos (Burkitt's lymphoma)	167

**Table 3:** Application of aptamers in clinical trials

Aptamers	Targets	Phase	References
Macugen	VEGF	Approved	168
E10030	PDGF	Phase III	169
AS1411	Nucleolin	Phase II	124
ARC1779	vWF	Phase II	170
NU172	Thrombin	Phase II	171
NOX-A12	SDF-1	Phase II	172
NOX-E36	MCP-1	Phase II	172
NOX-H94	Hepcidin	Phase I	173
ARC1905	Complement component 5 (C5)	Phase II	174
REG1	Coagulation factor IXa	Phase III	175
ARC19499	Hemophilia	Phase I	176

**Table 4:** Aptamer-based nonodrug delivery systems on targeted therapy of cancer cells

Aptamers	Nanomaterials	Target	Drugs	References
AS1411	Carbon nanotubes	Nucleolin (gastric cancer)	DOX	177
AS1411	PLGA NPs	Nucleolin (lung cancer)	Gemcitabine	178
EpCAM	Silica NPs	EpCAM (colon cancer)	DOX	179
A10-3-J1	Super paramagnetic iron oxide NPs	PSMA (prostate cancer)	DOX	180
Sgc8c	Gold NPs	PTK7 (leukemia)	Daunorubicin	181
TSA14	Liposome	TUBO breast cell line	DOX	182
5TR1	PLGA NPs	MUC1 (breast cancer)	Epirubicin	183
Endo28	RNA NPs	Annexin A2 (ovarian cancer); EGFR (cervical cancer)	DOX	184
EGFR	Albumin NPs	PSMA (prostate cancer)	Cisplatin	185
A10	PLGA NPs		TFO	186

## Abbreviations

SELEX: Systematic Evolution of Ligands by Exponential Enrichment
CTCs: circulating tumor cells
PCR: polymerase chain reaction
ChIP: chromatin immunoprecipitation
NECEEM: non-equilibrium capillary electrophoresis of equilibrium mixtures
PEG: polyethylene glycol
PSMA: prostate-specific membrane antigen
EpCAM: epithelial cell adhesion molecule
PTK7: protein tyrosine kinase 7
MUC1: mucin1
SERS: surface enhanced raman scattering
IgE: immunoglobulin E
FITC: fluorescein isothiocyanate
FRET: fluorescence resonance energy transfer
FETs: field-effect transistors
EIS: electrical impedance spectroscopy
PGDF: platelet-derived growth factor
BSA: albumin from bovine serum
CT: computed tomography
MRI: magnetic resonance imaging
PET: positron emission tomography
SPECT: single photon emission computed tomography
VEGFR2: vascular endothelial growth factor receptor 2
IHC: immunohistochemical
FDA: food and drug administration
NF-κB: nuclear factor-κB
AptDC: aptamer-drug conjugates
EPR: enhanced permeability and retention
PLGA: polylactic acid-glycolic acid copolymer
PTT: photothermal therapy
HCC: hepatocellular carcinoma
TILs: tumor-infiltrating lymphocytes
NK: nature killer
PD-1: programmed cell death 1
CTLA-4: cytotoxic T-lymphocyte associated protein 4
HER2: Erb-B2 receptor tyrosine kinase 2
Dox: doxorubicine
Bcl2: BCL2 apoptosis regulator
Plk1: polo like kinase 1

## References

[1] Bray F, Ferlay J, Soerjomataram I, Siegel RL, Torre LA, Jemal A (2018). Global cancer statistics 2018: GLOBOCAN estimates of incidence and mortality worldwide for 36 cancers in 185 countries. CA: a cancer journal for clinicians.

[2] Siegel RL, Miller KD, Jemal A (2018). Cancer statistics, 2018. CA: a cancer journal for clinicians.

[3] Vermes A, Guchelaar HJ, Dankert J (2000). Flucytosine: a review of its pharmacology, clinical indications, pharmacokinetics, toxicity and drug interactions. The Journal of antimicrobial chemotherapy.

[4] Reuther T, Schuster T, Mende U, Kubler A (2003). Osteoradionecrosis of the jaws as a side effect of radiotherapy of head and neck tumour patients-a report of a thirty year retrospective review. International journal of oral and maxillofacial surgery.

[5] Smith RA, Andrews KS, Brooks D, Fedewa SA, Manassaram-Baptiste D, Saslow D (2018). Cancer screening in the United States, 2018: A review of current American Cancer Society guidelines and current issues in cancer screening. CA: a cancer journal for clinicians.

[6] Bruno JG (2015). Predicting the Uncertain Future of Aptamer-Based Diagnostics and Therapeutics. Molecules.

[7] Tuerk C, Gold L (1990). Systematic evolution of ligands by exponential enrichment: RNA ligands to bacteriophage T4 DNA polymerase. Science.

[8] Osborne SE, Matsumura I, Ellington AD (1997). Aptamers as therapeutic and diagnostic reagents: problems and prospects. Current opinion in chemical biology.

[9] Mayer G (2009). The chemical biology of aptamers. Angewandte Chemie.

[10] Gelinas AD, Davies DR, Janjic N (2016). Embracing proteins: structural themes in aptamer-protein complexes. Current opinion in structural biology.

[11] Xi Z, Huang R, Deng Y, He N (2014). Progress in selection and biomedical applications of aptamers. Journal of biomedical nanotechnology.

[12] Ma H, Liu J, Ali MM, Mahmood MA, Labanieh L, Lu M (2015). Nucleic acid aptamers in cancer research, diagnosis and therapy. Chemical Society reviews.

[13] Xuan W, Peng Y, Deng Z, Peng T, Kuai H, Li Y (2018). A basic insight into aptamer-drug conjugates (ApDCs). Biomaterials.

[14] Hirao I, Kimoto M, Lee KH (2018). DNA aptamer generation by ExSELEX using genetic alphabet expansion with a mini-hairpin DNA stabilization method. Biochimie.

[15] Rothlisberger P, Hollenstein M (2018). Aptamer chemistry. Advanced drug delivery reviews.

[16] Bagalkot V, Farokhzad OC, Langer R, Jon S (2006). An aptamer-doxorubicin physical conjugate as a novel targeted drug-delivery platform. Angewandte Chemie.

[17] Farokhzad OC, Cheng J, Teply BA, Sherifi I, Jon S, Kantoff PW (2006). Targeted nanoparticle-aptamer bioconjugates for cancer chemotherapy *in vivo*. Proceedings of the National Academy of Sciences of the United States of America.

[18] McNamara JO 2nd, Andrechek ER, Wang Y, Viles KD, Rempel RE, Gilboa E (2006). Cell type-specific delivery of siRNAs with aptamer-siRNA chimeras. Nature biotechnology.

[19] Borzabadi-Farahani A, Borzabadi E, Lynch E (2014). Nanoparticles in orthodontics, a review of antimicrobial and anti-caries applications. Acta odontologica Scandinavica.

[20] Maeda H, Wu J, Sawa T, Matsumura Y, Hori K (2000). Tumor vascular permeability and the EPR effect in macromolecular therapeutics: a review. Journal of controlled release: official journal of the Controlled Release Society.

[21] Fang J, Nakamura H, Maeda H (2011). The EPR effect: Unique features of tumor blood vessels for drug delivery, factors involved, and limitations and augmentation of the effect. Advanced drug delivery reviews.

[22] Wieser A (2012). Review of reconstruction of radiation incident air kerma by measurement of absorbed dose in tooth enamel with EPR. Radiation protection dosimetry.

[23] Tian J, Ding L, Ju H, Yang Y, Li X, Shen Z (2014). A multifunctional nanomicelle for real-time targeted imaging and precise near-infrared cancer therapy. Angewandte Chemie.

[24] Hua X, Zhou Z, Yuan L, Liu S (2013). Selective collection and detection of MCF-7 breast cancer cells using aptamer-functionalized magnetic beads and quantum dots based nano-bio-probes. Analytica chimica acta.

[25] Niazi JH, Verma SK, Niazi S, Qureshi A (2015). *In vitro* HER2 protein-induced affinity dissociation of carbon nanotube-wrapped anti-HER2 aptamers for HER2 protein detection. The Analyst.

[26] Eikrem OS, Strauss P, Beisland C, Scherer A, Landolt L, Flatberg A (2016). Development and confirmation of potential gene classifiers of human clear cell renal cell carcinoma using next-generation RNA sequencing. Scandinavian journal of urology.

[27] Ellington AD, Szostak JW (1990). *In vitro* selection of RNA molecules that bind specific ligands. Nature.

[28] Mascini M, Palchetti I, Tombelli S (2012). Nucleic acid and peptide aptamers: fundamentals and bioanalytical aspects. Angewandte Chemie.

[29] Stoltenburg R, Reinemann C, Strehlitz B (2007). SELEX-a (r)evolutionary method to generate high-affinity nucleic acid ligands. Biomolecular engineering.

[30] Radom F, Jurek PM, Mazurek MP, Otlewski J, Jelen F (2013). Aptamers: molecules of great potential. Biotechnology advances.

[31] Caroli J, Taccioli C, De La Fuente A, Serafini P, Bicciato S (2016). APTANI: a computational tool to select aptamers through sequence-structure motif analysis of HT-SELEX data. Bioinformatics.

[32] Hermann T, Patel DJ (2000). Adaptive recognition by nucleic acid aptamers. Science.

[33] Shum KT, Zhou J, Rossi JJ (2013). Aptamer-based therapeutics: new approaches to combat human viral diseases. Pharmaceuticals.

[34] Martinez O, Bellard E, Golzio M, Mechiche-Alami S, Rols MP, Teissie J (2014). Direct validation of aptamers as powerful tools to image solid tumor. Nucleic acid therapeutics.

[35] Reinemann C, Strehlitz B (2014). Aptamer-modified nanoparticles and their use in cancer diagnostics and treatment. Swiss medical weekly.

[36] Keefe AD, Pai S, Ellington A (2010). Aptamers as therapeutics. Nature reviews Drug discovery.

[37] Wilson DS, Szostak JW (1999). *In vitro* selection of functional nucleic acids. Annual review of biochemistry.

[38] Duan N, Wu S, Chen X, Huang Y, Xia Y, Ma X (2013). Selection and characterization of aptamers against Salmonella typhimurium using whole-bacterium Systemic Evolution of Ligands by Exponential Enrichment (SELEX). Journal of agricultural and food chemistry.

[39] Dhar S, Gu FX, Langer R, Farokhzad OC, Lippard SJ (2008). Targeted delivery of cisplatin to prostate cancer cells by aptamer functionalized Pt(IV) prodrug-PLGA-PEG nanoparticles. Proceedings of the National Academy of Sciences of the United States of America.

[40] Bayat P, Nosrati R, Alibolandi M, Rafatpanah H, Abnous K, Khedri M (2018). SELEX methods on the road to protein targeting with nucleic acid aptamers. Biochimie.

[41] Klussmann S, Nolte A, Bald R, Erdmann VA, Furste JP (1996). Mirror-image RNA that binds D-adenosine. Nature biotechnology.

[42] Martell RE, Nevins JR, Sullenger BA (2002). Optimizing aptamer activity for gene therapy applications using expression cassette SELEX. Molecular therapy: the journal of the American Society of Gene Therapy.

[43] Vater A, Jarosch F, Buchner K, Klussmann S (2003). Short bioactive Spiegelmers to migraine-associated calcitonin gene-related peptide rapidly identified by a novel approach: tailored-SELEX. Nucleic acids research.

[44] Huang CJ, Lin HI, Shiesh SC, Lee GB (2010). Integrated microfluidic system for rapid screening of CRP aptamers utilizing systematic evolution of ligands by exponential enrichment (SELEX). Biosensors & bioelectronics.

[45] Lu J, Ho DM, Vogelaar NJ, Kraml CM, Pascal RA Jr (2004). A pentacene with a 144 degrees twist. Journal of the American Chemical Society.

[46] Drabovich A, Berezovski M, Krylov SN (2005). Selection of smart aptamers by equilibrium capillary electrophoresis of equilibrium mixtures (ECEEM). Journal of the American Chemical Society.

[47] Berezovski M, Musheev M, Drabovich A, Krylov SN (2006). Non-SELEX selection of aptamers. Journal of the American Chemical Society.

[48] Wen JD, Gray DM (2004). Selection of genomic sequences that bind tightly to Ff gene 5 protein: primer-free genomic SELEX. Nucleic acids research.

[49] White R, Rusconi C, Scardino E, Wolberg A, Lawson J, Hoffman M (2001). Generation of species cross-reactive aptamers using "toggle" SELEX. Molecular therapy: the journal of the American Society of Gene Therapy.

[50] Golden MC, Collins BD, Willis MC, Koch TH (2000). Diagnostic potential of PhotoSELEX-evolved ssDNA aptamers. Journal of biotechnology.

[51] Cox JC, Hayhurst A, Hesselberth J, Bayer TS, Georgiou G, Ellington AD (2002). Automated selection of aptamers against protein targets translated *in vitro*: from gene to aptamer. Nucleic acids research.

[52] Cerchia L, de Franciscis V (2010). Targeting cancer cells with nucleic acid aptamers. Trends in biotechnology.

[53] Camorani S, Cerchia L (2015). Oligonucleotide aptamers for glioma targeting: an update. Central nervous system agents in medicinal chemistry.

[54] Sefah K, Shangguan D, Xiong X, O'Donoghue MB, Tan W (2010). Development of DNA aptamers using Cell-SELEX. Nature protocols.

[55] Tan W, Donovan MJ, Jiang J (2013). Aptamers from cell-based selection for bioanalytical applications. Chemical reviews.

[56] Keefe AD, Cload ST (2008). SELEX with modified nucleotides. Current opinion in chemical biology.

[57] Cheung CH, Sun X, Kanwar JR, Bai JZ, Cheng L, Krissansen GW (2010). A cell-permeable dominant-negative survivin protein induces apoptosis and sensitizes prostate cancer cells to TNF-alpha therapy. Cancer cell international.

[58] Lin Y, Nieuwlandt D, Magallanez A, Feistner B, Jayasena SD (1996). High-affinity and specific recognition of human thyroid stimulating hormone (hTSH) by *in vitro*-selected 2'-amino-modified RNA. Nucleic acids research.

[59] Kujau MJ, Wolfl S (1998). Intramolecular derivatization of 2'-amino-pyrimidine modified RNA with functional groups that is compatible with re-amplification. Nucleic acids research.

[60] Ruckman J, Green LS, Beeson J, Waugh S, Gillette WL, Henninger DD (1998). 2'-Fluoropyrimidine RNA-based aptamers to the 165-amino acid form of vascular endothelial growth factor (VEGF165). Inhibition of receptor binding and VEGF-induced vascular permeability through interactions requiring the exon 7-encoded domain. The Journal of biological chemistry.

[61] Rusconi CP, Scardino E, Layzer J, Pitoc GA, Ortel TL, Monroe D (2002). RNA aptamers as reversible antagonists of coagulation factor IXa. Nature.

[62] Burmeister PE, Lewis SD, Silva RF, Preiss JR, Horwitz LR, Pendergrast PS (2005). Direct *in vitro* selection of a 2'-O-methyl aptamer to VEGF. Chemistry & biology.

[63] Burmeister PE, Wang C, Killough JR, Lewis SD, Horwitz LR, Ferguson A (2006). 2'-Deoxy purine, 2'-O-methyl pyrimidine (dRmY) aptamers as candidate therapeutics. Oligonucleotides.

[64] Healy JM, Lewis SD, Kurz M, Boomer RM, Thompson KM, Wilson C (2004). Pharmacokinetics and biodistribution of novel aptamer compositions. Pharmaceutical research.

[65] Rusconi CP, Roberts JD, Pitoc GA, Nimjee SM, White RR, Quick G Jr (2004). Antidote-mediated control of an anticoagulant aptamer *in vivo*. Nature biotechnology.

[66] Boomer RM, Lewis SD, Healy JM, Kurz M, Wilson C, McCauley TG (2005). Conjugation to polyethylene glycol polymer promotes aptamer biodistribution to healthy and inflamed tissues. Oligonucleotides.

[67] Kanwar JR, Roy K, Kanwar RK (2011). Chimeric aptamers in cancer cell-targeted drug delivery. Critical reviews in biochemistry and molecular biology.

[68] Vandghanooni S, Eskandani M, Barar J, Omidi Y (2018). Bispecific therapeutic aptamers for targeted therapy of cancer: a review on cellular perspective. Journal of molecular medicine.

[69] Cho Y, Lee YB, Lee JH, Lee DH, Cho EJ, Yu SJ (2016). Modified AS1411 Aptamer Suppresses Hepatocellular Carcinoma by Up-Regulating Galectin-14. PloS one.

[70] Pastor F (2016). Aptamers: A New Technological Platform in Cancer Immunotherapy. Pharmaceuticals.

[71] Soldevilla MM, Villanueva H, Pastor F (2016). Aptamers: A Feasible Technology in Cancer Immunotherapy. Journal of immunology research.

[72] Lupold SE, Hicke BJ, Lin Y, Coffey DS (2002). Identification and characterization of nuclease-stabilized RNA molecules that bind human prostate cancer cells via the prostate-specific membrane antigen. Cancer research.

[73] Shieh YA, Yang SJ, Wei MF, Shieh MJ (2010). Aptamer-based tumor-targeted drug delivery for photodynamic therapy. ACS nano.

[74] Cao Z, Tong R, Mishra A, Xu W, Wong GC, Cheng J (2009). Reversible cell-specific drug delivery with aptamer-functionalized liposomes. Angewandte Chemie.

[75] Song Y, Zhu Z, An Y, Zhang W, Zhang H, Liu D (2013). Selection of DNA aptamers against epithelial cell adhesion molecule for cancer cell imaging and circulating tumor cell capture. Analytical chemistry.

[76] Xiang D, Shigdar S, Qiao G, Wang T, Kouzani AZ, Zhou SF (2015). Nucleic acid aptamer-guided cancer therapeutics and diagnostics: the next generation of cancer medicine. Theranostics.

[77] Zhu G, Hu R, Zhao Z, Chen Z, Zhang X, Tan W (2013). Noncanonical self-assembly of multifunctional DNA nanoflowers for biomedical applications. Journal of the American Chemical Society.

[78] Zhu G, Zheng J, Song E, Donovan M, Zhang K, Liu C (2013). Self-assembled, aptamer-tethered DNA nanotrains for targeted transport of molecular drugs in cancer theranostics. Proceedings of the National Academy of Sciences of the United States of America.

[79] Ferreira CS, Matthews CS, Missailidis S (2006). DNA aptamers that bind to MUC1 tumour marker: design and characterization of MUC1-binding single-stranded DNA aptamers. Tumour biology: the journal of the International Society for Oncodevelopmental Biology and Medicine.

[80] Chang CC, Wei SC, Wu TH, Lee CH, Lin CW (2013). Aptamer-based colorimetric detection of platelet-derived growth factor using unmodified gold nanoparticles. Biosensors & bioelectronics.

[81] Mackey D, Kelly E, Nooney R (2016). Modelling random antibody adsorption and immunoassay activity. Mathematical biosciences and engineering: MBE.

[82] Qu F, Lu H, Yang M, Deng C (2011). Electrochemical immunosensor based on electron transfer mediated by graphene oxide initiated silver enhancement. Biosensors & bioelectronics.

[83] Wei H, Wang E (2011). Electrochemiluminescence of tris(2,2'-bipyridyl)ruthenium and its applications in bioanalysis: a review. Luminescence: the journal of biological and chemical luminescence.

[84] Hu K, Yang H, Zhou J, Zhao S, Tian J (2013). Aptasensor for amplified IgE sensing based on fluorescence quenching by graphene oxide. Luminescence: the journal of biological and chemical luminescence.

[85] Hosseini M, Mehrabi F, Ganjali MR, Norouzi P (2016). A fluorescent aptasensor for sensitive analysis oxytetracycline based on silver nanoclusters. Luminescence: the journal of biological and chemical luminescence.

[86] Razmi N, Baradaran B, Hejazi M, Hasanzadeh M, Mosafer J, Mokhtarzadeh A (2018). Recent advances on aptamer-based biosensors to detection of platelet-derived growth factor. Biosensors & bioelectronics.

[87] Huang CC, Huang YF, Cao Z, Tan W, Chang HT (2005). Aptamer-modified gold nanoparticles for colorimetric determination of platelet-derived growth factors and their receptors. Analytical chemistry.

[88] Ye S, Zhai X, Wu Y, Kuang S (2016). Dual-primer self-generation SERS signal amplification assay for PDGF-BB using label-free aptamer. Biosensors & bioelectronics.

[89] Li H, Wang M, Wang C, Li W, Qiang W, Xu D (2013). Silver nanoparticle-enhanced fluorescence resonance energy transfer sensor for human platelet-derived growth factor-BB detection. Analytical chemistry.

[90] Jiang Y, Fang X, Bai C (2004). Signaling aptamer/protein binding by a molecular light switch complex. Analytical chemistry.

[91] Cao ZJ, Peng QW, Qiu X, Liu CY, Lu JZ (2011). Highly sensitive chemiluminescence technology for protein detection using aptamer-based rolling circle amplification platform. Journal of pharmaceutical analysis.

[92] Chai Y, Tian D, Gu J, Cui H (2011). A novel electrochemiluminescence aptasensor for protein based on a sensitive N-(aminobutyl)-N-ethylisoluminol-functionalized gold nanoprobe. The Analyst.

[93] Meirinho SG, Dias LG, Peres AM, Rodrigues LR (2016). Voltammetric aptasensors for protein disease biomarkers detection: A review. Biotechnology advances.

[94] Thevenot DR, Toth K, Durst RA, Wilson GS (2001). Electrochemical biosensors: recommended definitions and classification. Biosensors & bioelectronics.

[95] He L, Zhang S, Ji H, Wang M, Peng D, Yan F (2016). Protein-templated cobaltous phosphate nanocomposites for the highly sensitive and selective detection of platelet-derived growth factor-BB. Biosensors & bioelectronics.

[96] Chen W, Zheng R, Baade PD, Zhang S, Zeng H, Bray F (2016). Cancer statistics in China, 2015. CA: a cancer journal for clinicians.

[97] Siegel RL, Miller KD, Jemal A (2016). Cancer statistics, 2016. CA: a cancer journal for clinicians.

[98] Zhang X, Soori G, Dobleman TJ, Xiao GG (2014). The application of monoclonal antibodies in cancer diagnosis. Expert review of molecular diagnostics.

[99] Nagrath S, Sequist LV, Maheswaran S, Bell DW, Irimia D, Ulkus L (2007). Isolation of rare circulating tumour cells in cancer patients by microchip technology. Nature.

[100] Alix-Panabieres C, Pantel K (2016). Clinical Applications of Circulating Tumor Cells and Circulating Tumor DNA as Liquid Biopsy. Cancer discovery.

[101] Jacob K, Sollier C, Jabado N (2007). Circulating tumor cells: detection, molecular profiling and future prospects. Expert review of proteomics.

[102] Yoon HJ, Kim TH, Zhang Z, Azizi E, Pham TM, Paoletti C (2013). Sensitive capture of circulating tumour cells by functionalized graphene oxide nanosheets. Nature nanotechnology.

[103] Yu M, Bardia A, Wittner BS, Stott SL, Smas ME, Ting DT (2013). Circulating breast tumor cells exhibit dynamic changes in epithelial and mesenchymal composition. Science.

[104] Bi S, Ji B, Zhang Z, Zhang S (2013). A chemiluminescence imaging array for the detection of cancer cells by dual-aptamer recognition and bio-bar-code nanoprobe-based rolling circle amplification. Chemical communications.

[105] Kashefi-Kheyrabadi L, Mehrgardi MA, Wiechec E, Turner AP, Tiwari A (2014). Ultrasensitive detection of human liver hepatocellular carcinoma cells using a label-free aptasensor. Analytical chemistry.

[106] Zeng Z, Tung CH, Zu Y (2014). A cancer cell-activatable aptamer-reporter system for one-step assay of circulating tumor cells. Molecular therapy Nucleic acids.

[107] Wang K, Fan D, Liu Y, Wang E (2015). Highly sensitive and specific colorimetric detection of cancer cells via dual-aptamer target binding strategy. Biosensors & bioelectronics.

[108] Zhao W, Cui CH, Bose S, Guo D, Shen C, Wong WP (2012). Bioinspired multivalent DNA network for capture and release of cells. Proceedings of the National Academy of Sciences of the United States of America.

[109] Sheng W, Chen T, Tan W, Fan ZH (2013). Multivalent DNA nanospheres for enhanced capture of cancer cells in microfluidic devices. ACS nano.

[110] Massoud TF, Paulmurugan R, Gambhir SS (2004). Molecular imaging of homodimeric protein-protein interactions in living subjects. FASEB journal: official publication of the Federation of American Societies for Experimental Biology.

[111] Shi H, Tang Z, Kim Y, Nie H, Huang YF, He X (2010). *In vivo* fluorescence imaging of tumors using molecular aptamers generated by cell-SELEX. Chemistry, an Asian journal.

[112] Zhong H, Zhang Q, Zhang S (2011). High-intensity fluorescence imaging and sensitive electrochemical detection of cancer cells by using an extracellular supramolecular reticular DNA-quantum dot sheath. Chemistry.

[113] Rockey WM, Huang L, Kloepping KC, Baumhover NJ, Giangrande PH, Schultz MK (2011). Synthesis and radiolabeling of chelator-RNA aptamer bioconjugates with copper-64 for targeted molecular imaging. Bioorganic & medicinal chemistry.

[114] Yu MK, Kim D, Lee IH, So JS, Jeong YY, Jon S (2011). Image-guided prostate cancer therapy using aptamer-functionalized thermally cross-linked superparamagnetic iron oxide nanoparticles. Small.

[115] Zeng Z, Parekh P, Li Z, Shi ZZ, Tung CH, Zu Y (2014). Specific and sensitive tumor imaging using biostable oligonucleotide aptamer probes. Theranostics.

[116] Wu X, Zhao Z, Bai H, Fu T, Yang C, Hu X (2015). DNA Aptamer Selected against Pancreatic Ductal Adenocarcinoma for *in vivo* Imaging and Clinical Tissue Recognition. Theranostics.

[117] Ma H, Gao Z, Yu P, Shen S, Liu Y, Xu B (2014). A dual functional fluorescent probe for glioma imaging mediated by blood-brain barrier penetration and glioma cell targeting. Biochemical and biophysical research communications.

[118] Kim B, Yang J, Hwang M, Choi J, Kim HO, Jang E (2013). Aptamer-modified magnetic nanoprobe for molecular MR imaging of VEGFR2 on angiogenic vasculature. Nanoscale research letters.

[119] Heo D, Lee E, Ku M, Hwang S, Kim B, Park Y (2014). Maleimidyl magnetic nanoplatform for facile molecular MRI. Nanotechnology.

[120] Zeng Z, Zhang P, Zhao N, Sheehan AM, Tung CH, Chang CC (2010). Using oligonucleotide aptamer probes for immunostaining of formalin-fixed and paraffin-embedded tissues. Modern pathology: an official journal of the United States and Canadian Academy of Pathology, Inc.

[121] Li X, An Y, Jin J, Zhu Z, Hao L, Liu L (2015). Evolution of DNA aptamers through *in vitro* metastatic-cell-based systematic evolution of ligands by exponential enrichment for metastatic cancer recognition and imaging. Analytical chemistry.

[122] Zhang Z, Ali MM, Eckert MA, Kang DK, Chen YY, Sender LS (2013). A polyvalent aptamer system for targeted drug delivery. Biomaterials.

[123] Ng EW, Shima DT, Calias P, Cunningham ET Jr, Guyer DR, Adamis AP (2006). Pegaptanib, a targeted anti-VEGF aptamer for ocular vascular disease. Nature reviews Drug discovery.

[124] Bates PJ, Laber DA, Miller DM, Thomas SD, Trent JO (2009). Discovery and development of the G-rich oligonucleotide AS1411 as a novel treatment for cancer. Experimental and molecular pathology.

[125] Mongelard F, Bouvet P (2010). AS-1411, a guanosine-rich oligonucleotide aptamer targeting nucleolin for the potential treatment of cancer, including acute myeloid leukemia. Current opinion in molecular therapeutics.

[126] Darisipudi MN, Kulkarni OP, Sayyed SG, Ryu M, Migliorini A, Sagrinati C (2011). Dual blockade of the homeostatic chemokine CXCL12 and the proinflammatory chemokine CCL2 has additive protective effects on diabetic kidney disease. The American journal of pathology.

[127] Duda DG, Kozin SV, Kirkpatrick ND, Xu L, Fukumura D, Jain RK (2011). CXCL12 (SDF1alpha)-CXCR4/CXCR7 pathway inhibition: an emerging sensitizer for anticancer therapies?. Clinical cancer research: an official journal of the American Association for Cancer Research.

[128] Bates PJ, Kahlon JB, Thomas SD, Trent JO, Miller DM (1999). Antiproliferative activity of G-rich oligonucleotides correlates with protein binding. The Journal of biological chemistry.

[129] Soundararajan S, Chen W, Spicer EK, Courtenay-Luck N, Fernandes DJ (2008). The nucleolin targeting aptamer AS1411 destabilizes Bcl-2 messenger RNA in human breast cancer cells. Cancer research.

[130] Mahlknecht G, Maron R, Mancini M, Schechter B, Sela M, Yarden Y (2013). Aptamer to ErbB-2/HER2 enhances degradation of the target and inhibits tumorigenic growth. Proceedings of the National Academy of Sciences of the United States of America.

[131] Parekh P, Kamble S, Zhao N, Zeng Z, Portier BP, Zu Y (2013). Immunotherapy of CD30-expressing lymphoma using a highly stable ssDNA aptamer. Biomaterials.

[132] Wu X, Chen J, Wu M, Zhao JX (2015). Aptamers: active targeting ligands for cancer diagnosis and therapy. Theranostics.

[133] Thiel KW, Hernandez LI, Dassie JP, Thiel WH, Liu X, Stockdale KR (2012). Delivery of chemo-sensitizing siRNAs to HER2+-breast cancer cells using RNA aptamers. Nucleic acids research.

[134] Dai F, Zhang Y, Zhu X, Shan N, Chen Y (2012). Anticancer role of MUC1 aptamer-miR-29b chimera in epithelial ovarian carcinoma cells through regulation of PTEN methylation. Targeted oncology.

[135] Liu M, Yu X, Chen Z, Yang T, Yang D, Liu Q (2017). Aptamer selection and applications for breast cancer diagnostics and therapy. Journal of nanobiotechnology.

[136] Zhao N, Pei SN, Qi J, Zeng Z, Iyer SP, Lin P (2015). Oligonucleotide aptamer-drug conjugates for targeted therapy of acute myeloid leukemia. Biomaterials.

[137] Huang YF, Shangguan D, Liu H, Phillips JA, Zhang X, Chen Y (2009). Molecular assembly of an aptamer-drug conjugate for targeted drug delivery to tumor cells. Chembiochem: a European journal of chemical biology.

[138] Subramanian N, Raghunathan V, Kanwar JR, Kanwar RK, Elchuri SV, Khetan V (2012). Target-specific delivery of doxorubicin to retinoblastoma using epithelial cell adhesion molecule aptamer. Molecular vision.

[139] Liu Z, Duan JH, Song YM, Ma J, Wang FD, Lu X (2012). Novel HER2 aptamer selectively delivers cytotoxic drug to HER2-positive breast cancer cells *in vitro*. Journal of translational medicine.

[140] Rink JS, Plebanek MP, Tripathy S, Thaxton CS (2013). Update on current and potential nanoparticle cancer therapies. Current opinion in oncology.

[141] Xiao Z, Farokhzad OC (2012). Aptamer-functionalized nanoparticles for medical applications: challenges and opportunities. ACS nano.

[142] Yang L, Zhang X, Ye M, Jiang J, Yang R, Fu T (2011). Aptamer-conjugated nanomaterials and their applications. Advanced drug delivery reviews.

[143] Zhang L, Radovic-Moreno AF, Alexis F, Gu FX, Basto PA, Bagalkot V (2007). Co-delivery of hydrophobic and hydrophilic drugs from nanoparticle-aptamer bioconjugates. ChemMedChem.

[144] Xing H, Tang L, Yang X, Hwang K, Wang W, Yin Q (2013). Selective Delivery of an Anticancer Drug with Aptamer-Functionalized Liposomes to Breast Cancer Cells *in vitro* and *in vivo*. Journal of materials chemistry B.

[145] Xiao Z, Levy-Nissenbaum E, Alexis F, Luptak A, Teply BA, Chan JM (2012). Engineering of targeted nanoparticles for cancer therapy using internalizing aptamers isolated by cell-uptake selection. ACS nano.

[146] Tong GJ, Hsiao SC, Carrico ZM, Francis MB (2009). Viral capsid DNA aptamer conjugates as multivalent cell-targeting vehicles. Journal of the American Chemical Society.

[147] Sun H, Zu Y (2015). Aptamers and their applications in nanomedicine. Small.

[148] Huang YF, Sefah K, Bamrungsap S, Chang HT, Tan W (2008). Selective photothermal therapy for mixed cancer cells using aptamer-conjugated nanorods. Langmuir: the ACS journal of surfaces and colloids.

[149] Pala K, Serwotka A, Jelen F, Jakimowicz P, Otlewski J (2014). Tumor-specific hyperthermia with aptamer-tagged superparamagnetic nanoparticles. International journal of nanomedicine.

[150] Borghaei H, Paz-Ares L, Horn L, Spigel DR, Steins M, Ready NE (2015). Nivolumab versus Docetaxel in Advanced Nonsquamous Non-Small-Cell Lung Cancer. The New England journal of medicine.

[151] Mohri K, Nishikawa M, Takahashi N, Shiomi T, Matsuoka N, Ogawa K (2012). Design and development of nanosized DNA assemblies in polypod-like structures as efficient vehicles for immunostimulatory CpG motifs to immune cells. ACS nano.

[152] Liu H, Kwong B, Irvine DJ (2011). Membrane anchored immunostimulatory oligonucleotides for *in vivo* cell modification and localized immunotherapy. Angewandte Chemie.

[153] Gilboa E, McNamara J 2nd, Pastor F (2013). Use of oligonucleotide aptamer ligands to modulate the function of immune receptors. Clinical cancer research: an official journal of the American Association for Cancer Research.

[154] Gilboa E, Berezhnoy A, Schrand B (2015). Reducing Toxicity of Immune Therapy Using Aptamer-Targeted Drug Delivery. Cancer immunology research.

[155] Herrmann A, Priceman SJ, Swiderski P, Kujawski M, Xin H, Cherryholmes GA (2014). CTLA4 aptamer delivers STAT3 siRNA to tumor-associated and malignant T cells. The Journal of clinical investigation.

[156] Zhang D, Zheng Y, Lin Z, Lan S, Zhang X, Zheng A (2019). Artificial Engineered Natural Killer Cells Combined with Antiheat Endurance as a Powerful Strategy for Enhancing Photothermal-Immunotherapy Efficiency of Solid Tumors. Small.

[157] Gros A, Robbins PF, Yao X, Li YF, Turcotte S, Tran E (2014). PD-1 identifies the patient-specific CD8(+) tumor-reactive repertoire infiltrating human tumors. The Journal of clinical investigation.

[158] Prodeus A, Abdul-Wahid A, Fischer NW, Huang EH, Cydzik M, Gariepy J (2015). Targeting the PD-1/PD-L1 Immune Evasion Axis With DNA Aptamers as a Novel Therapeutic Strategy for the Treatment of Disseminated Cancers. Molecular therapy Nucleic acids.

[159] Shigdar S, Lin J, Yu Y, Pastuovic M, Wei M, Duan W (2011). RNA aptamer against a cancer stem cell marker epithelial cell adhesion molecule. Cancer science.

[160] Shigdar S, Qiao L, Zhou SF, Xiang D, Wang T, Li Y (2013). RNA aptamers targeting cancer stem cell marker CD133. Cancer letters.

[161] Bayat P, Taghdisi SM, Rafatpanah H, Abnous K, Ramezani M (2019). *In vitro* selection of CD70 binding aptamer and its application in a biosensor design for sensitive detection of SKOV-3 ovarian cells. Talanta.

[162] Almasi F, Mousavi Gargari SL, Bitaraf F, Rasoulinejad S (2016). Development of a Single Stranded DNA Aptamer as a Molecular Probe for LNCap Cells Using Cell-SELEX. Avicenna journal of medical biotechnology.

[163] Daniels DA, Chen H, Hicke BJ, Swiderek KM, Gold L (2003). A tenascin-C aptamer identified by tumor cell SELEX: systematic evolution of ligands by exponential enrichment. Proceedings of the National Academy of Sciences of the United States of America.

[164] Alshaer W, Hillaireau H, Vergnaud J, Ismail S, Fattal E (2015). Functionalizing Liposomes with anti-CD44 Aptamer for Selective Targeting of Cancer Cells. Bioconjugate chemistry.

[165] Haghighi FH, Binaymotlagh R, Mirahmadi-Zare SZ, Hadadzadeh H (2020). Aptamer/magnetic nanoparticles decorated with fluorescent gold nanoclusters for selective detection and collection of human promyelocytic leukemia (HL-60) cells from a mixture. Nanotechnology.

[166] Fafinska J, Czech A, Sitz T, Ignatova Z, Hahn U (2018). DNA Aptamers for the Malignant Transformation Marker CD24. Nucleic acid therapeutics.

[167] Mallikaratchy P, Tang Z, Kwame S, Meng L, Shangguan D, Tan W (2007). Aptamer directly evolved from live cells recognizes membrane bound immunoglobin heavy mu chain in Burkitt's lymphoma cells. Molecular & cellular proteomics: MCP.

[168] Vinores SA (2006). Pegaptanib in the treatment of wet, age-related macular degeneration. International journal of nanomedicine.

[169] Jaffe GJ, Ciulla TA, Ciardella AP, Devin F, Dugel PU, Eandi CM (2017). Dual Antagonism of PDGF and VEGF in Neovascular Age-Related Macular Degeneration: A Phase IIb, Multicenter, Randomized Controlled Trial. Ophthalmology.

[170] Spiel AO, Mayr FB, Ladani N, Wagner PG, Schaub RG, Gilbert JC (2009). The aptamer ARC1779 is a potent and specific inhibitor of von Willebrand Factor mediated *ex vivo* platelet function in acute myocardial infarction. Platelets.

[171] Buff MC, Schafer F, Wulffen B, Muller J, Potzsch B, Heckel A (2010). Dependence of aptamer activity on opposed terminal extensions: improvement of light-regulation efficiency. Nucleic acids research.

[172] Vater A, Klussmann S (2015). Turning mirror-image oligonucleotides into drugs: the evolution of Spiegelmer((R)) therapeutics. Drug discovery today.

[173] van Eijk LT, John AS, Schwoebel F, Summo L, Vauleon S, Zollner S (2014). Effect of the antihepcidin Spiegelmer lexaptepid on inflammation-induced decrease in serum iron in humans. Blood.

[174] Leung E, Landa G (2013). Update on current and future novel therapies for dry age-related macular degeneration. Expert review of clinical pharmacology.

[175] Povsic TJ, Vavalle JP, Alexander JH, Aberle LH, Zelenkofske SL, Becker RC (2014). Use of the REG1 anticoagulation system in patients with acute coronary syndromes undergoing percutaneous coronary intervention: results from the phase II RADAR-PCI study. EuroIntervention: journal of EuroPCR in collaboration with the Working Group on Interventional Cardiology of the European Society of Cardiology.

[176] Waters EK, Genga RM, Schwartz MC, Nelson JA, Schaub RG, Olson KA (2011). Aptamer ARC19499 mediates a procoagulant hemostatic effect by inhibiting tissue factor pathway inhibitor. Blood.

[177] Taghavi S, Nia AH, Abnous K, Ramezani M (2017). Polyethylenimine-functionalized carbon nanotubes tagged with AS1411 aptamer for combination gene and drug delivery into human gastric cancer cells. International journal of pharmaceutics.

[178] Alibolandi M, Ramezani M, Abnous K, Hadizadeh F (2016). AS1411 Aptamer-Decorated Biodegradable Polyethylene Glycol-Poly(lactic-co-glycolic acid) Nanopolymersomes for the Targeted Delivery of Gemcitabine to Non-Small Cell Lung Cancer *In vitro*. Journal of pharmaceutical sciences.

[179] Xie X, Li F, Zhang H, Lu Y, Lian S, Lin H (2016). EpCAM aptamer-functionalized mesoporous silica nanoparticles for efficient colon cancer cell-targeted drug delivery. European journal of pharmaceutical sciences: official journal of the European Federation for Pharmaceutical Sciences.

[180] Leach JC, Wang A, Ye K, Jin S (2016). A RNA-DNA Hybrid Aptamer for Nanoparticle-Based Prostate Tumor Targeted Drug Delivery. International journal of molecular sciences.

[181] Taghdisi SM, Danesh NM, Lavaee P, Emrani AS, Hassanabad KY, Ramezani M (2016). Double targeting, controlled release and reversible delivery of daunorubicin to cancer cells by polyvalent aptamers-modified gold nanoparticles. Materials science & engineering C, Materials for biological applications.

[182] Moosavian SA, Abnous K, Badiee A, Jaafari MR (2016). Improvement in the drug delivery and anti-tumor efficacy of PEGylated liposomal doxorubicin by targeting RNA aptamers in mice bearing breast tumor model. Colloids and surfaces B, Biointerfaces.

[183] Taghavi S, Ramezani M, Alibolandi M, Abnous K, Taghdisi SM (2017). Chitosan-modified PLGA nanoparticles tagged with 5TR1 aptamer for *in vivo* tumor-targeted drug delivery. Cancer letters.

[184] Pi F, Zhang H, Li H, Thiviyanathan V, Gorenstein DG, Sood AK (2017). RNA nanoparticles harboring annexin A2 aptamer can target ovarian cancer for tumor-specific doxorubicin delivery. Nanomedicine: nanotechnology, biology, and medicine.

[185] Chen Y, Wang J, Wang J, Wang L, Tan X, Tu K (2016). Aptamer Functionalized Cisplatin-Albumin Nanoparticles for Targeted Delivery to Epidermal Growth Factor Receptor Positive Cervical Cancer. Journal of biomedical nanotechnology.

[186] Jiao J, Zou Q, Zou MH, Guo RM, Zhu S, Zhang Y (2016). Aptamer-modified PLGA nanoparticle delivery of triplex forming oligonucleotide for targeted prostate cancer therapy. Neoplasma.

